# The Inhibitory Effect of Wheat Husks Addition on Aflatoxins Production by *Aspergillus flavus* in Liquid Culture With Various Wheat Compositions as Carbon Sources

**DOI:** 10.3389/fmicb.2020.01448

**Published:** 2020-07-15

**Authors:** Khaled M. Ghanem, Walid A. Lotfy, Mohamed M. El-Shaer, Samy A. Elassar

**Affiliations:** ^1^Department of Botany and Microbiology, Faculty of Science, Alexandria University, Alexandria, Egypt; ^2^Department of Microbiology, Faculty of Dentistry, Pharos University in Alexandria, Alexandria, Egypt

**Keywords:** *Aspergillus flavus*, aflatoxins, wheat, response surface methodology, fractional factorial design

## Abstract

Wheat may be infected by the aflatoxigenic mold *Aspergillus flavus* during pre- and post-harvest activities. Control strategies reported to manage aflatoxin contamination of wheat are expensive and require extensive testing to verify the absence of toxic secondary metabolites or newly formed compounds. The objective of this study was to develop an *in vitro* new control strategy based on assessing the influence of wheat husks on aflatoxin production by *A. flavus* in liquid culture. The results showed that aflatoxin production is significantly influenced by the existence of husks in the wheat forms used as carbon substrates according to the following order: full wheat grains < half-crushed wheat grains < wheat flour 82% < wheat flour 72%. By applying a fractional factorial design and a response surface methodology, maximum aflatoxin production (2.567 ng/mg) was predicted when wheat flour 72% (39 g/l) as a carbon source, yeast extract (5 g/l), and a 75-ml medium volume/250 ml flask were utilized. At this optimized condition, after addition of wheat husk extract, the growth and synthesis of aflatoxins of *A. flavus* were repressed by 74.85 and 98.72%, respectively. This finding paves the way to examine the antifungal potential of wheat husk constituents and to compare their efficacy with thyme, cinnamon, sweet basil, and coriander essential oils, which possess antimycotic activities. Accordingly, the wheat husk component SiO_2_ showed the highest growth inhibition (67.04%) and reduction of *A. flavus* aflatoxins (82.67%). These results are comparable to those obtained from various examined antimycotic essential oils.

## Introduction

Wheat and wheat products are the main food for four billion people and constitute a major part of the daily human and animal diet. The Food and Agriculture Organization of the United Nations (FAO) estimates the annual world production of wheat in 2015 at approximately 2540 million tons (Food and Agriculture Organization of the United Nations [FAO], 2016).

Mycotoxins are poisonous secondary metabolites produced by fungi, which are considered one of the most important risks associated with wheat consumption, leading to an annual loss of $932 million in stored grains (Cheli et al., 2017). Aflatoxins B_1_ (AFB_1_) and B_2_ (AFB_2_), produced typically by the virulent *Aspergillus flavus*, are the most serious mycotoxins affecting wheat (Bennett and Klich, 2003; Ayalew, 2010; Cheli et al., 2013). Aflatoxins are favorably produced during post-harvest activities, such as transportation and storage, due to poor hygienic conditions, high temperature, and moisture content and heavy rains (Hell et al., 2000; Van Egmond and Jonker, 2004; Cheli et al., 2017), which may result in yield reduction of wheat products due to deterioration of wheat grains (Siuda et al., 2010). Furthermore, aflatoxins have hepatotoxic, mutagenic, immunosuppressive, teratogenic, and carcinogenic effects on humans and animals (Bennett and Klich, 2003).

Many strategies, including physical, chemical, and biological control, have been investigated to manage aflatoxin contamination of wheat (Fandohan et al., 2005; Ni and Streett, 2005; Yin et al., 2008). However, chemical or physical treatment adds to the cost of wheat already damaged from fungal growth. Moreover, extensive testing is required to ascertain that a new compound with a different mode of action has not been formed. Whereas, biological control may pose a potential contamination of wheat with toxic secondary metabolites (Dorner, 2004). On the other hand, wheat husks contain silicon (Terzioǧlu and Yucel, 2012), which is reported to control fungal plant pathogens (Sakr, 2016). Therefore, the aim of the present work is to develop an *in vitro* new preventive management of aflatoxin production by *A. flavus* based on the addition of wheat husks to liquid culture. The latter condition was chosen to provide accelerated fungal growth and to support a manageable comprehensive study of various factors affecting the production of aflatoxins. A fractional factorial design and a response surface methodology were adopted to achieve an optimized maximum production of aflatoxins by *A. flavus*. This optimized condition was used to evaluate the antifungal potential of wheat husks and wheat husk constituents compared to thyme, cinnamon, sweet basil, and coriander essential oils, which possess antimycotic activities (Mokbel and Alharbi, 2015; Chahal et al., 2016; Khorasani et al., 2017; Debonne et al., 2018). This investigation is important for providing the necessary *in vitro* knowledge to develop an *in vivo* study for a sustainable and cost-effective control of aflatoxins. To the best of our knowledge, this method has not been reported for aflatoxins control so far.

## Materials and Methods

### Wheat Samples

One of the most commonly cultivated and productive wheat cultivar was selected, namely Sods 12. A total of 16 wheat samples were collected from farms in the Delta region, Egypt. The samples were maintained in plastic bags in a dark, cold (4°C), and dry place for not more than 1 month until analysis.

### Chemicals

All chemicals used throughout this study were of analytical grade except for methanol that was of HPLC grade. All chemicals were purchased from Himedia Laboratories (Mumbai, India). Kits used for aflatoxin determination were purchased from VICAM (A Water Business, Milford, DE, United States).

### Culture Media

Dehydrated culture media used throughout this work were purchased from Oxoid (England) or Lab M (England). All media were prepared and sterilized according to the manufacturer’s instructions.

Sabouraud dextrose (SD) agar: peptone, 10 g/l; dextrose, 40 g/l; agar, 15 g/l. This medium was used to obtain heavy fungal sporulation that was used for seed inoculation.

Czapek yeast extract (CYE) broth: K_2_HPO_4_, 1 g/l; yeast extract, 5 g/l; sucrose, 30 g/l; Czapek concentrate (ml/l), 10. This medium was used for aflatoxin production; under some specified experiments, sucrose was replaced by various forms of wheat.

Czapek concentrate: NaNO_3_, 0.3 g/l; KCl, 0.05 g/l; MgSO_4_.7H_2_O, 0.05 g/l; FeSO_4_.7H_2_O, 0.001 g/l; ZnSO_4_.7H_2_O, 0.001 g/l; CuSO_4_.5H_2_O, 0.0005 g/l.

### Preparation of Wheat Samples

Five kilograms of dried wheat spikes were collected; the weight of husks per kilogram of wheat spikes was 34.3 g. Full wheat grains were classified into four categories by means of sieving as follows: full wheat grains; half-crushed wheat grains of particle size 3 mm using a woven wire mesh sieve; flour 82%, wheat flour of particle size 0.16 mm using a nylon mesh sieve followed by adding bran residue after sieving; and flour 72%, wheat flour of particle size 0.16 mm using a nylon mesh sieve without bran fine. Samples with moisture content between 8 and 14% were maintained in airtight plastic bags in a refrigerator at 4°C until analysis as reported in Sisman and Ergin (2011).

### Preparation of Seed Inoculum

The strain used throughout this work was *A. flavus* ATCC 16883. Suspension of spores was prepared by culturing *A. flavus* on SD agar for 5 days and then washing the culture with sterile saline solution (0.9% NaCl). Spore suspension was mixed for 1 min before counting the number of spores by a hemocytometer to adjust the count to the desired concentration.

### Production of Aflatoxins by *A. flavus*

*Aspergillus flavus* was cultivated in an aliquot of 50 ml CYE broth medium/250 ml Erlenmeyer flask. The medium contained 30 g/l of various wheat forms, one at a time, as a sole carbon source. It was important to avoid the differences in solubility of wheat forms; therefore, the obtained aflatoxin concentrations were standardized based on the residual total concentration of fermentable sugars in each carbon source (Miller, 1959). The incubation periods were adjusted as indicated in the Results section. After incubation at 28°C in a shaking incubator at 180 rpm for the specified incubation period, the growth was determined as dry weight, and total aflatoxins were estimated by using a VICAM fluorometer.

### Quantitative Determination of Aflatoxins

Total concentration of aflatoxins (B1, B2, G1, and G2) was measured by using immunoaffinity columns (Aflatest system, VICAM) and fluorometer (VICAM series 4EX) according to the instructions of the manufacturer. Briefly, 25 ml of mycelia-free broth were mixed in blender jar containing 200 ml methanol:water (80:20 v/v) for 1 min. The extract was filtered through Whatman filter paper (Cat No. 1001125), and an aliquot of 10 ml of filtrate was transferred into a falcon tube and was diluted with 20 ml distilled water and then filtered through 1.5 μm glass microfiber (No. 31955, VICAM). An aliquot of 1 ml of filtered extract was transferred to the immunoaffinity column headspace for filtration at a flow rate of one drop/s by adjusting the speed of the pump (No. 20650 VICAM). Elution was performed by addition of 1 ml methanol, and the eluate was received into a glass cuvette. One milliliter of aflatest developer solution was added to the final eluate in the cuvette. A standard curve was established for quantification of total aflatoxins using VICAM fluorometer. The method of aflatoxins determination was described by the manufacturer and validated before determination (validation parameters: regression = 0.99988, limit of detection = 0.001 ng/mg, limit of quantification = 0.00303 ng/mg and relative standard deviation = 0.725%).

### Determination of Fungal Dry Weight

An aliquot of 3 ml of the broth medium containing mycelia was filtered through a predried (at 60°C) and preweighed filter paper. The filter cake was washed with 20 ml distilled water and dried for 48 h at 60°C. The filter cake was allowed to cool in a desiccator and weighed (Lotfy et al., 2007).

### The Effect of Various Wheat Forms on the Production of Aflatoxins by *A. flavus*

*Aspergillus flavus* was grown on CYE broth containing various forms of wheat grains as carbon sources, one at a time, and aflatoxin production (ng/mg) was monitored at different incubation times (0, 5, 10, 15, 20, 21, and 25 days).

### Elucidation of the Factors Affecting Aflatoxin Production by *A. flavus*

To maximize the production of aflatoxins by *A. flavus*, a fraction factorial design, the Plackett-Burman design (Plackett and Burman, 1946; Lotfy et al., 2017), and Box-Behnken design, a response surface methodology, were applied (Box and Behnken, 1960; Lotfy et al., 2018). The purpose of first statistical design was to identify which variable has a significant effect on aflatoxin production by *A. flavus*. In this experiment, seven variables were screened in eight experimental runs, the selected variables included yeast extract, carbon source (various forms of wheat), Czapek concentrate, K_2_HPO_4_, in addition to inoculum size, medium volume/flask, and pH. For each variable, high (+) and low (−) levels were tested, and each trial was performed in triplicate. The main effect and the significance of each variable were determined using Statistica 10 software. The main effect was calculated according to the following equation:

$${\text{E}}_{\text{xi}}=\left(\sum {\text{M}}_{\text{i}+}-\sum {\text{M}}_{i-}\right)/\text{N}$$

where E_*xi*_ is the variable main effect, M_*i+*_ and M_*i*__–_ are the aflatoxin production in trials where the independent variable (xi) was present in high and low concentrations, respectively, and N is the number of trials divided by two. A positive main effect sign indicates that a high level of this variable is nearer to maximum response, and a negative sign indicates that the low level of this variable is nearer to maximum response. An aliquot of 50 ml of CYE broth was used as the basal culture medium, and sucrose was replaced by various forms of wheat. The inoculum size used was 2 ml of 6 × 10^5^ spores/ml, and the pH was adjusted at 7.5 with incubation for 21 days at 28°C under shaking at 180 rpm. To describe the nature of the response surface in the experimental region and to reveal the optimal levels of the most significant independent variables, the Box-Behnken design was applied. The most significant independent variables concluded from the Plackett-Burman experiment were yeast extract g/l (*X1*), carbon source g/l (*X2*), and medium volume ml (*X3*). Accordingly, these variables were tested at three levels (low, basal, and high) coded (−1, 0, and +1) in 15 treatment combinations for predicting the optimal level. The following second order polynomial model was fitted to correlate relationship between independent variables and response:

$$\begin{array}{c} Y={\text{b}}_{0}+{\text{b}}_{1}X_{1}+{\text{b}}_{2}X_{2}+{\text{b}}_{3}X_{3}+{\text{b}}_{12}X_{1}X_{2}+{\text{b}}_{13}X_{1}X_{3}\\ \;+{\text{b}}_{23}X_{2}X_{3}+{\text{b}}_{11}X_{1}^{2}+{\text{b}}_{22}X_{2}^{2}+{\text{b}}_{33}X_{3}^{2} \end{array}$$

where *Y* is the dependent variable (aflatoxin production in ng/mg); *X1*, *X2*, and *X3* are the levels of the independent variables; b_0_ is the regression coefficient at the center point; b_1_, b_2_, and b_3_ are linear coefficients; b_12_, b_13_, and b_23_ are the second order interaction coefficients; and b_11_, b_22_, and b_33_ are quadratic coefficients. The values of the coefficients were calculated and optimal levels were predicted using Statistica 10 software. The coefficient of determination *R*^2^ was selected to reflect the quality of the model fitting.

### Effect of Wheat Husks and Wheat Husk Constituents on Aflatoxin Production by *A. flavus*

The optimized condition obtained by the Box-Behnken experiment (model 4) was conducted, and the liquid cultures were autoclaved at 121°C for 15 min and then inoculated with 6 × 10^5^ *A. flavus* spores/ml. Various essential oils, namely thyme, cinnamon, sweet basil, and coriander oils, were added one at a time at a concentration of 1000 ppm. In addition, individual components of wheat husks, viz. silicon dioxide, potassium oxide, calcium oxide, magnesium oxide, and ferric oxide, were added one at a time at a concentration equivalent to each individual component ratio in full wheat grains. An extract of 5% (w/w) ground wheat husks was also added to the optimal medium, and a control was conducted under the same conditions without addition of oils or chemicals. After incubation for 21 days at 28°C, the fungal dry weight and aflatoxin production were assayed. Chemical analysis of wheat husks and the tested essential oils were carried out at Chemistry Laboratories Administration, Egypt using FT-IR spectrometer (Nicolet iS5, Thermo Fisher) equipped with an iD7 accessory in the range of 560–3200 nm. A calibration curve was constructed for quantification of each analyte using various concentrations of the standard chemical.

### Statistical Analysis

Experiments were carried out in triplicate, and the mean and standard deviation were computed. A statistical significance difference was set at a *P*-value of 0.05. Response surface regression, which is a form of multivariate non-linear regression, was adopted.

## Results

### The Effect of Various Wheat Forms on the Production of Aflatoxins by *A. flavus*

**FIGURE 1:**
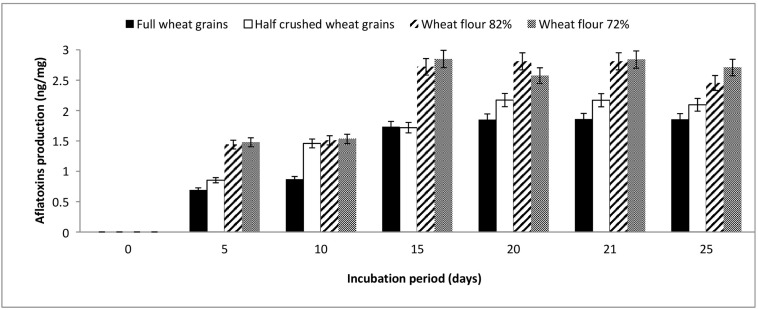
Production of aflatoxins (ng/mg) by *A. flavus* grown on various forms of wheat grains and wheat flour at different incubation periods.

The results illustrated in Figure 1 show that full and half-crushed wheat grains demonstrated aflatoxin levels lower than flour 82 and 72% during the entire monitoring period (5–25 days). Aflatoxin production by *A. flavus* ranged from 0.69–1.86, 0.85–2.17, 1.43–2.8, and 1.47–2.85 ng/mg when grown on full wheat grains, half-crushed wheat grains, flour 82% and flour 72%, respectively. At the end of the incubation period, the mycosynthesis of aflatoxins by *A. flavus* grown on flour 82 and 72% was higher than aflatoxins produced on full wheat grains and half-crushed wheat grains by approximately 132.2, 117.16, 145.88, and 129.28%, respectively. On the other hand, the optimal incubation time was 21 days for aflatoxin production by *A. flavus* grown on the examined wheat forms.

### Elucidation of the Factors Affecting Aflatoxin Production by *A. flavus*

**FIGURE 2:**
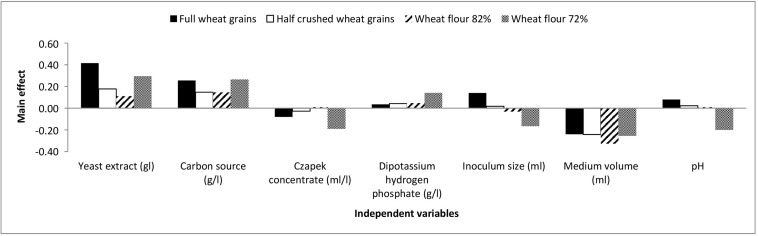
The main effects of independent variables on aflatoxins production (ng/mg) by *A. flavus* grown on various forms of wheat grains and wheat flour based on the Plackett-Burman experiment.

The chosen levels of the Plackett-Burman design variables and observations are presented in Supplementary Table S1. The main effects of each variable with respect to aflatoxin formation by *A. flavus* grown on full wheat grains, half-crushed wheat grains, flour 82% and flour 72% are presented in the Supplementary Material and are illustrated graphically in Figure 2.

Based on the results of the Plackett-Burman experiment, a near optimum condition for maximal aflatoxin production is as follows: yeast extract, 8 g/l; carbon source, 48 g/l; Czapek concentrate, 4 ml/l; K_2_HPO_4_, 1.6 g/l; inoculum size (6 × 10^5^ spores/ml), 1 ml; medium volume, 25/250 ml Erlenmeyer flask; and medium pH, 4.5 with an incubation period of 21 days at 28°C under shaking at 180 rpm.

Moreover, a significant effect (*p* ≤ 0.05) of yeast extract (g/l), carbon source (g/l), and medium volume (ml)/250 ml Erlenmeyer flask on aflatoxin production (ng/mg) by *A. flavus* was perceived. Consequently, the Box-Behnken design was adopted to determine the most favorable level of these factors relating to aflatoxin synthesis by *A. flavus*. As shown in Supplementary Table S2, the design matrix includes 15 trials, and each factor was examined at three different levels (−, 0, and +). The observed and predicted values of aflatoxins (ng/mg) produced by *A. flavus* on full wheat grains, half-crushed wheat grains, flour 82%, and flour 72% are shown in Supplementary Table S2. The values of *R*^2^ are 0.955, 0.968, 0.998, and 0.996 for the four models of aflatoxin synthesis (ng/mg) by *A. flavus* on full wheat grains, half-crushed wheat grains, flour 82%, and flour 72%, respectively. The values of *R*^2^ for the predicted data of the four models are 0.966, 0.971, 0.999, and 0.998, respectively. These values indicate a high precision of the four models and a reliable degree of fitting between predicted and observed data.

**FIGURE 3:**
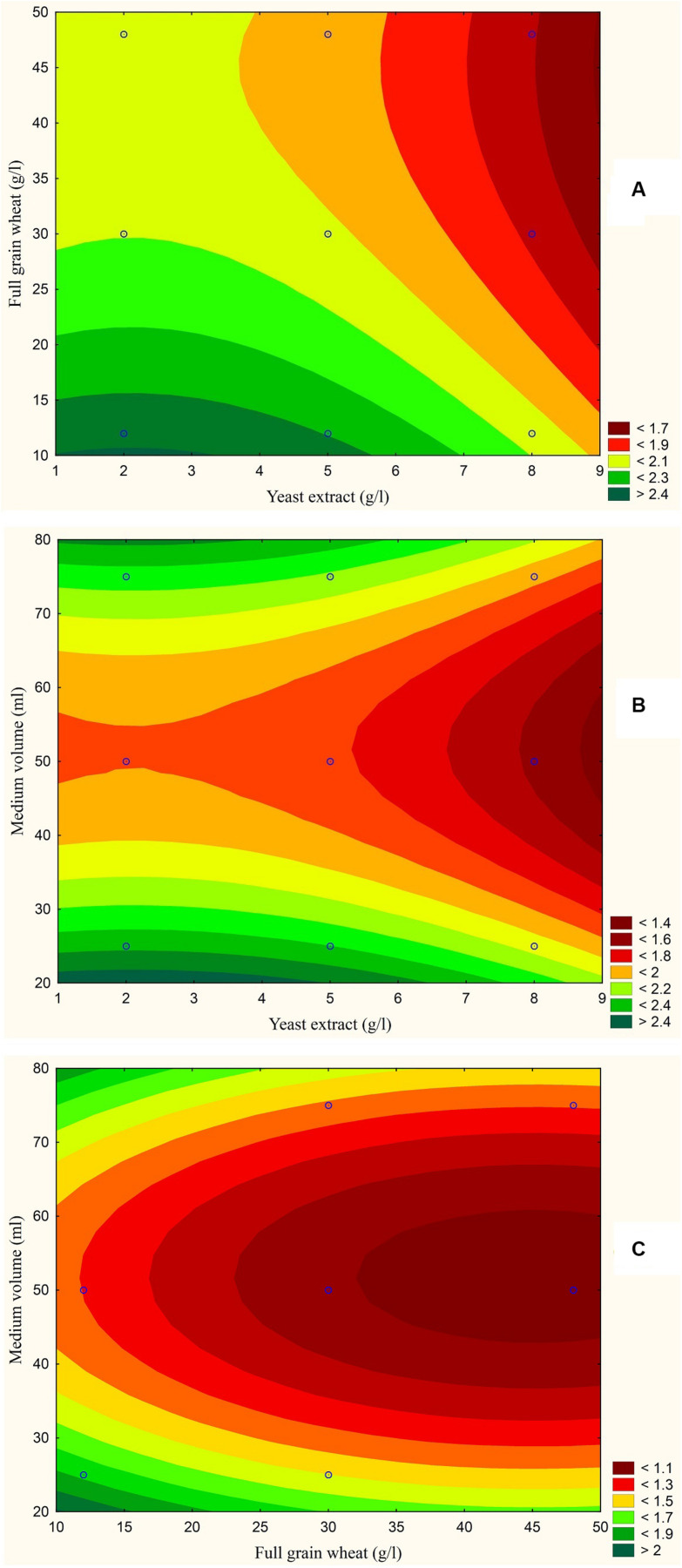
Response contour plot based on the Box-Behnken experimental results. **(A)** The combined effect of yeast extract (g/l) and full wheat grains (g/l) on aflatoxin production (ng/mg). **(B)** The combined effect of yeast extract (g/l) and medium volume (ml) on aflatoxin production (ng/mg). **(C)** The combined effect of full wheat grains (g/l) and medium volume (ml) on aflatoxin production (ng/mg).

**FIGURE 4:**
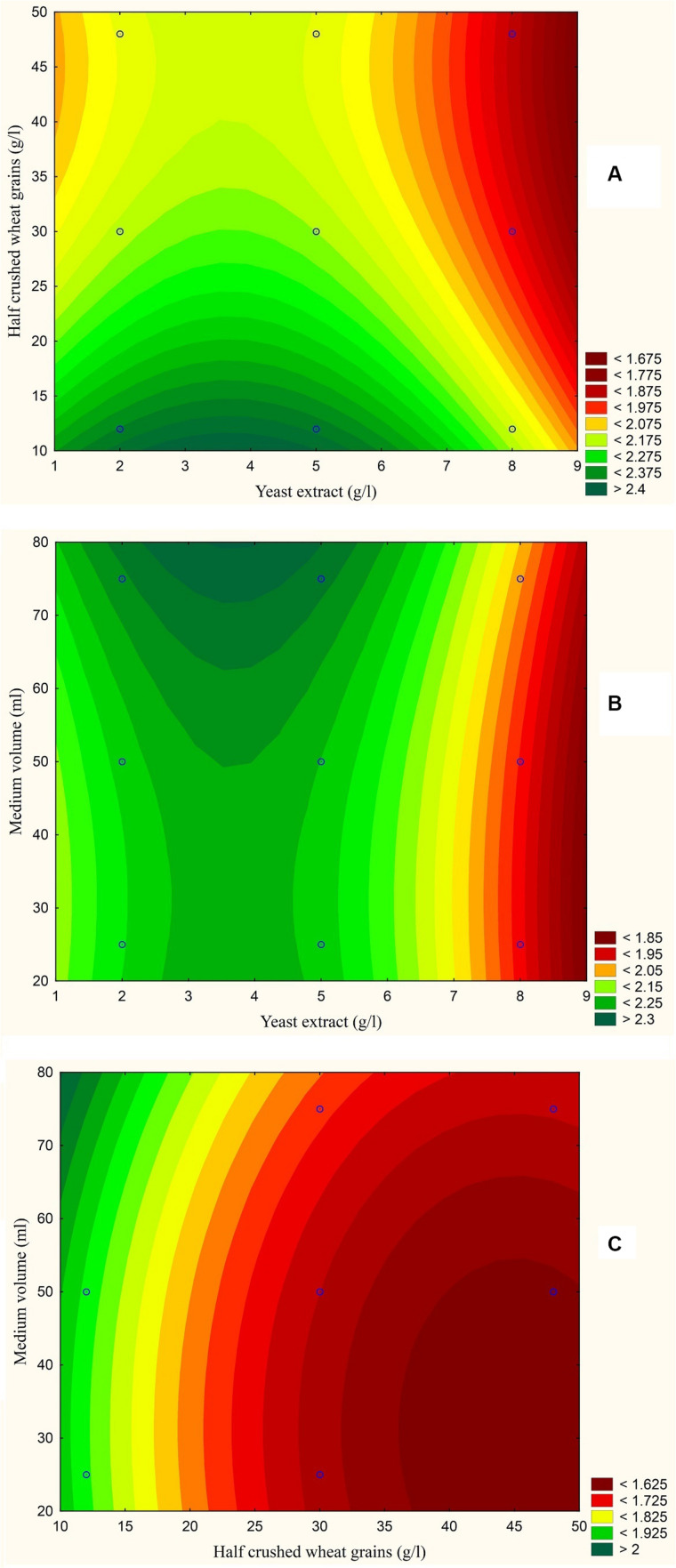
Response contour plot based on the Box-Behnken experimental results. **(A)** The combined effect of yeast extract (g/l) and half-crushed wheat grains (g/l) on aflatoxin production (ng/mg). **(B)** The combined effect of yeast extract (g/l) and medium volume (ml) on aflatoxin production (ng/mg). **(C)** The combined effect of half-crushed wheat grains (g/l) and medium volume (g/l) on aflatoxin production (ng/mg).

**FIGURE 5:**
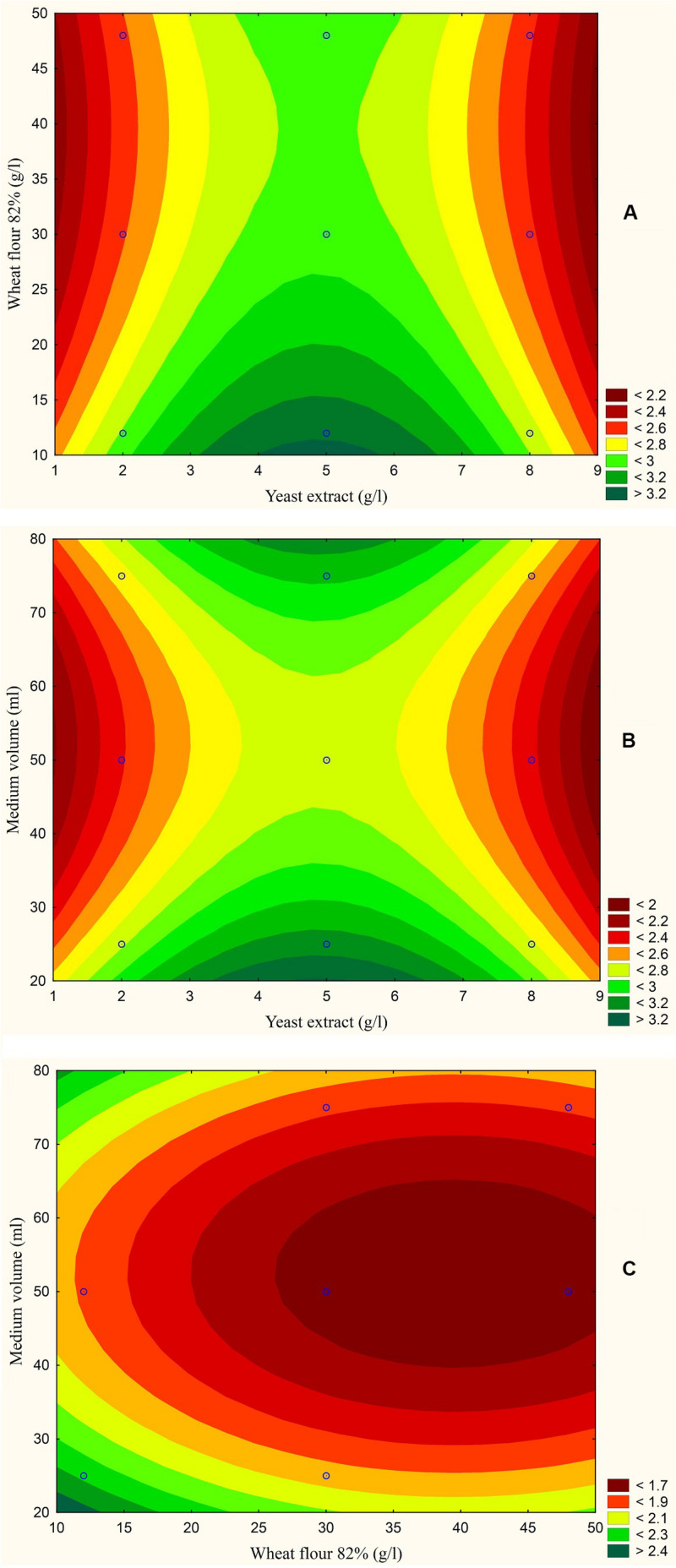
Response contour plot based on the Box-Behnken experimental results. **(A)** The combined effect of yeast extract (g/l) and wheat flour 82% (g/l) on aflatoxin production (ng/mg). **(B)** The combined effect of yeast extract (g/l) and medium volume (ml) on aflatoxin production (ng/mg). **(C)** The combined effect of wheat flour 82% (g/l) and medium volume (ml) on aflatoxin production (ng/mg).

**FIGURE 6:**
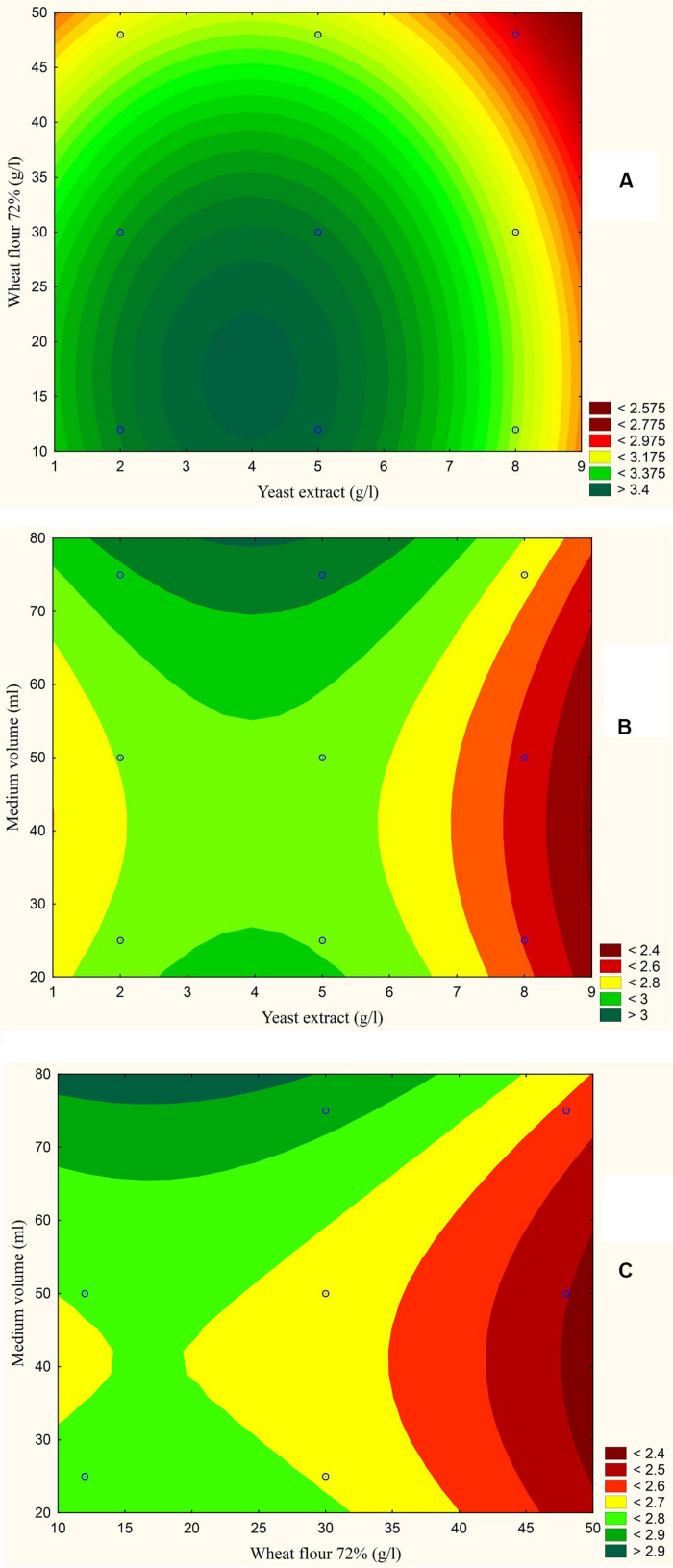
Response contour plot based on the Box-Behnken experimental results. **(A)** The combined effect of yeast extract (g/l) and wheat flour 72% (g/l) on aflatoxin production (ng/mg). **(B)** The combined effect of yeast extract (g/l) and medium volume (ml) on aflatoxin production (ng/mg). **(C)** The combined effect of wheat flour 72% (g/l) and medium volume (g/l) on aflatoxin production (ng/mg).

The results are also presented graphically as illustrated in Figures 3–6 to generate a contour plot of the polynomial equations. Figures 3–6 describe the effects of yeast extract (g/l), various forms of wheat as carbon sources (g/l), and medium volume (ml)/250 ml Erlenmeyer flask on the production of aflatoxins (ng/mg) by *A. flavus*. The production of aflatoxins is clearly elevated at 2 g/l yeast extract, 10 g/l full wheat grains, and 20 ml medium volume (Figure 3); 4 g/l yeast extract, 10 g/l half-crushed wheat grains, and 80 ml medium volume (Figure 4); 4 g/l yeast extract, 10 g/l wheat flour 82%, and 20 ml medium volume (Figure 5); and 5 g/l yeast extract, 30 g/l wheat flour 72%, and 80 ml medium volume (Figure 6).

The statistical analyses of the experiment (Supplementary Table S3) were performed, and second order polynomial functions were generated with predicted maximum aflatoxin production by *A. flavus* (ng/mg) (Table 1).

**TABLE 1:** Predicted maximum aflatoxin production by *A. flavus* (ng/mg) and equations of the quadratic models based on Box-Behnken experiment.

Model	Carbon source	Carbon source (g/l)	Yeast extract (g/l)	Medium volume (ml)	Predicted aflatoxins production by *A. flavus* (ng/mg)
1	Full wheat grains	12	2	25	1.247
2	Half crushed wheat grains	12	3.5	75	1.647
3	Wheat flour 82%	12	5	25	1.822
4	Wheat flour 72%	39	5	75	2.567
Model 1 function	*Y* = 0.63 − 0.36*X*_1_ − 0.027*X*_2_ + 0.07*X*_3_ + 0.03*X_1_^2^* + 0.0003*X_2_^2^* − 0.0007*X_3_^2^* + 0.0162*X_1_X_2_* − 0.0004*X_1_X_3_* − 0.0001*X_2_X_3_*				
Model 2 function	*Y* = 0.942 + 0.217*X*_1_ + 0.028*X*_2_ + 0.0075*X*_3_ − 0.0189*X_1_^2^* − 0.0081*X_1_X_2_* − 0.0005*X_1_X_3_* − 0.0001*X_2_X_3_*				
Model 3 function	*Y* = 0.2259 − 0.092*X*_1_ + 0.115*X*_2_ + 0.0575*X*_3_ + 0.0262*X_1_^2^* − 0.0016*X_2_^2^* − 0.0005*X_3_^2^* − 0.0191*X_1_X_2_* − 0.0002*X_1_X_3_* + 0.0002*X_2_X_3_*				
Model 4 function	*Y* = 2.159 + 0.1403*X*_1_ + 0.0223*X*_2_ + 0.0135*X*_3_ − 0.0145*X_1_^2^* − 0.0001*X_2_^2^* − 0.0002*X_3_^2^* − 0.0136*X_1_X_2_* + 0.0004*X_1_X_3_* + 0.0001*X_2_X_3_*				

### Effect of Wheat Husk Addition on Aflatoxin Production by *A. flavus* Compared to Various Essential Oils

**FIGURE 7:**
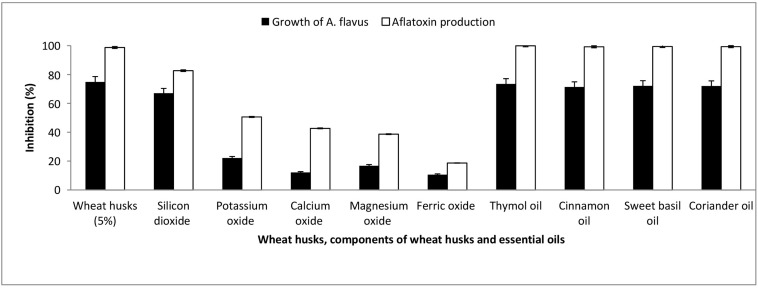
The inhibitory effect of wheat husks, wheat husk components, and various essential oils on the growth and aflatoxin production of *A. flavus*.

The previous finding may infer that wheat husks have a potential antimycotic activity against *A. flavus*, which results in diminished production of aflatoxin. In order to assess the effect of wheat husk addition on aflatoxin production by *A. flavus*, 5% (w/w) ground wheat husk extract was added to the optimal medium revealed in model four. Interestingly, after incubation for 21 days at 28°C under shaking at 180 rpm, the addition of wheat husk extract inhibited the growth and aflatoxin formation of *A. flavus* by 74.85 and 98.72%, respectively (Figure 7).

The chemical analysis of Sods 12 wheat husk cultivar revealed that SiO_2_ (50.3%), K_2_O (10.7%), CaO (7.5%), MgO (1.3%), and Fe_2_O_3_ (1.01%) were the major components. Figure 7 shows the inhibitory effect of SiO_2_, K_2_O, CaO, MgO, and Fe_2_O_3_ on aflatoxin production and growth of *A. flavus*. Among the major components of wheat husks, SiO_2_ demonstrated the highest inhibitory effect against growth of *A. flavus* and aflatoxin production, 67.04% and 82.67%, respectively. On the other hand, Fe_2_O_3_ exhibited the lowest antagonistic effect toward *A. flavus* growth and aflatoxin production, 10.06% and 18.67%, respectively.

Thymol, cinnamon, sweet basil, and coriander essential oils were tested for their effect on the growth and aflatoxin production by *A. flavus* compared to wheat husks. Fifteen components representing 99.91% of thymol oil constituents were spectroscopically identified. The major components detected were p-cymene (8.41%), γ-terpinene (30.90%), and thymol (47.59%). Thymol oil strongly inhibited fungal growth and aflatoxin production by 73.46 and 99.9%, respectively. Twenty components representing 99.97% of cinnamon oil constituents were spectroscopically determined. The major components were trans-Cinnamaldehyde (35.22%), Copaene (26.47%), α-Muurolene (8.21%), and α-Cadinene (9.79%). Cinnamon oil sharply repressed fungal growth and aflatoxin production by 71.35 and 99.2%, respectively. The chemical composition of sweet basil oil revealed 25 components representing 98.6% of the oil. The major components detected were methyl eugenol (39.3%) and methyl chavicol (38.3%). The antifungal activity of sweet basil essential oil against *A. flavus* growth recorded an inhibition of 72.11% while it showed 99.4% reduction in the fungus potentiality to produce aflatoxins. Ten components representing 99.53% of coriander oil constituents were identified, and the major component was linalool (89.46%). The coriander oil greatly inhibited fungal growth by 72%, and the production of aflatoxins by *A. flavus* was intensely reduced by 99.3%.

## Discussion

There are reports regarding the role of seed coat and its integrity on the aflatoxin production during storage (Hadavi, 2006); our results suggest that the same concept may be prevailing in wheat. On the other hand, physical, chemical, and biological control strategies of aflatoxin contamination are costly and require broad testing to confirm the absence of newly formed compounds or toxic secondary metabolites. The objective of the current study was to develop an *in vitro* new control strategy based on evaluating the influence of wheat husks on aflatoxin production by *A. flavus* in liquid culture. *A. flavus* grown on CYE broth containing full and half-crushed wheat grains as carbon sources demonstrated aflatoxin levels lower than flour 82 and 72%. The implication of this finding is that wheat husks in the full and half-crushed wheat grains affected the biosynthesis of aflatoxins by *A. flavus*.

*A. flavus* utilizes variable levels of carbon and nitrogen sources and inorganic substances for growth and aflatoxin production in crops (Mellon et al., 2005; Rajasekaran et al., 2017). Therefore, the Plackett-Burman and Box-Behnken designs were adopted to determine the most favorable levels of environmental factors affecting the growth and aflatoxin synthesis of *A. flavus*. It was noticeable that full wheat grains supported the lowest amount of aflatoxins produced by *A. flavus*. Moreover, aflatoxin production followed the next descending order: full wheat grains < half-crushed wheat grains < wheat flour 82% < wheat flour 72%. It can be supposed that these data are due to the higher presence of wheat husks in full grains and not to the readily accessible carbohydrates in milled wheat flour rather than wheat kernels. This is simply because aflatoxin production was determined in terms of specific productivity, aflatoxins (ng)/fungal dry weight (mg).

The results of the chemical analysis of wheat husks are in keeping with the findings reported by Terzioǧlu and Yucel (2012): SiO_2_ (43.22%), K_2_O (11.3%), CaO (5.46%), MgO (0.99%), and Fe_2_O_3_ (0.84%). SiO_2_ demonstrated the highest inhibitory effect against *A. flavus* growth and production of aflatoxins, which may be attributed to the ability of silicon dioxide to interrupt cell functions, such as cell differentiation (Dizaj et al., 2014). Conversely, Fe_2_O_3_ exhibited the lowest antagonistic effect toward the growth of *A. flavus* and production of aflatoxins, which may be due to inducing the decomposition of proteins and lipopolysaccharides in the fungal cell membrane (Parveen et al., 2018). Fe_2_O_3_ can also cause oxidative damage to the fungal cells via penetration through disrupted membranes (Parveen et al., 2018). On the other hand, magnesium oxide, calcium oxide, and potassium oxide showed a moderate inhibitory effect on both fungal growth and aflatoxin synthesis by *A. flavus*. Calcium oxide and magnesium oxide act as potent antifungal agents due to the generation of active oxygen species and alkalinity, which damage the cell membrane leading to the leakage of intracellular contents (Yamamoto et al., 2010). As previously proposed (Ortega-Aguilar et al., 2011) potassium oxide was reported to reduce sporulation, modify the colony growth patterns of fungi, and affect mycelial growth.

An attempt was performed to compare the antifungal and antiaflatoxigenic activities of wheat husks to some essential oils that possess antimycotic properties. Thymol oil strongly inhibited fungal growth and aflatoxin production, which was attributed to the presence of phenolic compounds, thymol, and terpene hydrocarbons, γ-terpinene, with a synergistic effect (Dorman and Deans, 2000; Skočibušić et al., 2006; Rota et al., 2008; Gallucci et al., 2009). Other studies suggest another synergistic effect of the minor components of thymol oil in relation to its antifungal activity (Gill et al., 2002). Cinnamon oil sharply repressed the growth of *A. flavus* and aflatoxin production owing to the presence of cinnamaldehyde, which inhibits spore germination and improves lipid peroxidation and oxidative stress in *A. flavus* (Sun et al., 2016). Our results clearly demonstrate that sweet basil oil showed inhibitory effects against growth and aflatoxin production of *A. flavus*, which may be associated to the leakage of intracellular ATP and K^+^ by the phenolic components of the oil, leading to the cell’s death (Pandey et al., 2014). Coriander oil effectively inhibited aflatoxin production by *A. flavus* through linalool, which proved to change the functions of the fungal membrane protein leading to an electrolyte imbalance and death (Khan et al., 2010; De Oliveira Lima et al., 2017). Our study shows that, at an optimized condition, after addition of wheat husk extract, the antifungal and antiaflatoxigenic activities to *A. flavus* are comparable to those obtained from thymol, cinnamon, sweet basil, and coriander oils. There is a lack of published sources describing the repression mechanism of wheat husks on the biosynthesis of aflatoxins by *A. flavus*. Many authors reported that wheat husks efficiently protect wheat kernels from fungal infestation and their mycotoxins *in vivo*, but evaluation of aflatoxins is still scant (Infektion, 2004; Castoria et al., 2005; Suchowilska et al., 2010; Moudry et al., 2011; Vučković et al., 2013). Moheb et al. (2013) reported that wheat husks contain 770 ± 157 mg/kg dry weight of the flavone tricin, which possesses effective antifungal properties (Jiang et al., 2020). Therefore, the antifungal effect of wheat husks on *A. flavus* might be also attributed to the suppression of spore germination by tricin, which was previously reported on the fungal pathogen *Fusarium oxysporum* by Kong et al. (2010).

## Conclusion

Wheat husks showed significant antimycotic and antiaflatoxigenic activity against *A. flavus*. The addition of husks extract to wheat containing liquid culture as a sole carbon source drastically protected the wheat from aflatoxicosis and fungal growth. To our knowledge, this is the first report on the *in vitro* application of a natural waste for aflatoxin control by a simple and cost-effective method. However, further studies are recommended to investigate the *in vivo* addition of wheat husks to various grains or nuts liable to aflatoxicosis. It is also suggested to study the impact of wheat husks on expression of genes such as *aflR*, *aflJ*, *norA*, *omtA*, *omtB*, *pksA*, *vbs*, *ver-1*, and *hexA*, which regulate aflatoxin biosynthesis in *A. flavus*.

## Data Availability Statement

All datasets generated for this study are included in the article/Supplementary Material.

## Author Contributions

KG, SE, and WL designed the study and supervised all of the experimental works. ME-S carried out the experiments. WL analyzed the data and wrote the manuscript. All authors contributed to the article and approved the submitted version.

## Conflict of Interest

The authors declare that the research was conducted in the absence of any commercial or financial relationships that could be construed as a potential conflict of interest.
