# Supplementary material for: The Inhibitory Effect of Wheat Husks Addition on Aflatoxins Production by Aspergillus flavus in Liquid Culture With Various Wheat Compositions as Carbon Sources
Source: Front Microbiol. 2020 Jul 15;11:1448. doi: 10.3389/fmicb.2020.01448 (PMC7381238; doi:10.3389/fmicb.2020.01448)
Supplement: Supplementary file 1 [file Data_Sheet_1.pdf]

Supplementary Material

The main effects of each variable with respect to aflatoxins formation by *A. flavus* based on the Plackett-Burman design

Yeast extract (g/l), carbon source (g/l) and culture volume (ml) showed the highest values of main effect among the seven independent variables included in this study. Yeast extract (g/l), carbon source (g/l) and K_2HPO_4 (g/l) were found to have a positive main influence on aflatoxins formation whereas, medium volume (ml) revealed a negative main effect on aflatoxins production by *A. flavus*. On the other hand, the influences of Czapek concentrate (ml/l), inoculum size (ml) and medium pH on aflatoxins production by *A. flavus* were differed according to the type of wheat form used as a carbon source. Higher levels of Czapek concentrate (ml/l) in the medium containing flour 82% as a carbon source were ideal for enhancing aflatoxins formation while its lower levels in the media containing other wheat forms were favourable for increasing aflatoxins formation. Inoculum size (ml) showed a positive main effect values with respect to aflatoxins production by *A. flavus* grown on full wheat grains and half crushed wheat grains whereas, it showed a negative main effect when *A. flavus* was grown on flour 82% and flour 72%. Higher pH of the culture media containing full wheat grains, half crushed wheat grains and flour 82% as a carbon source was ideal for augmenting aflatoxins synthesis while its lower level in the media containing flour 72% was favourable for increasing aflatoxins formations.

Supplementary TABLE S1 Levels of independent variables affecting aflatoxins production by *A. flavus* and observations of the Plackett-Burman experiment.

Trial	Independent variable							Aflatoxins production (ng/mg)			
	Yeast extract (g/l)	Carbon source (g/l)	Czapek concentrate (ml/l)	Dipotassium hydrogen phosphate (g/l)	Inoculum size (ml)	Medium volume (ml)	pH	Full wheat grains	Half crushed wheat grains	Wheat flour 82%	Wheat flour 72%
**1**	2	12	4	1.6	3	75	4.5	1.63	1.66	2.21	2.50
**2**	8	12	4	0.4	1	75	7.5	1.95	1.80	2.31	2.62
**3**	2	48	4	0.4	3	25	7.5	2.17	2.03	2.64	2.68
**4**	8	48	4	1.6	1	25	4.5	2.40	2.21	2.82	3.48
**5**	2	12	16	1.6	1	25	7.5	1.73	1.88	2.58	2.53
**6**	8	12	16	0.4	3	25	4.5	2.17	2.01	2.61	2.72
**7**	2	48	16	0.4	1	75	4.5	1.63	1.72	2.35	2.60
**8**	8	48	16	1.6	3	75	7.5	2.30	1.98	2.48	2.67

Supplementary TABLE S2 Observations and predictions of aflatoxins production by *A. flavus* based on Box-Behnken experiment.

**Trial**	**Factor 1**	**Factor 2**	**Factor 3**	**Full wheat grains**		**Half crushed wheat grains**		**Wheat flour 82%**		**Wheat flour 72%**	
	X1 **Yeast extract (g/l)**	***X2*** **Carbon source (g/l)**	***X3*** **Medium volume (ml)**	**Observed Aflatoxins (ng/mg)**	**Predicted Aflatoxins (ng/mg)**	**Observed Aflatoxins (ng/mg)**	**Predicted Aflatoxins (ng/mg)**	**Observed Aflatoxins (ng/mg)**	**Predicted Aflatoxins (ng/mg)**	**Observed Aflatoxins (ng/mg)**	**Predicted Aflatoxins (ng/mg)**
**1**	2	12	50	1.85	2.29	1.71	1.38	2.47	1.69	2.70	1.45
**2**	8	12	50	2.01	2.05	1.90	1.25	2.70	3.42	2.71	2.21
**3**	2	48	50	2.08	2.04	2.11	2.76	2.82	2.09	2.81	3.31
**4**	8	48	50	2.30	1.86	2.29	2.61	3.15	3.92	3.45	4.7
**5**	2	30	75	1.57	1.42	1.98	1.97	2.80	4.22	2.59	2.86
**6**	8	30	75	1.95	2.2	2.19	2.5	2.62	2.54	2.81	2.33
**7**	2	30	25	1.47	1.22	1.85	1.54	2.58	2.65	2.68	3.15
**8**	8	30	25	1.98	2.13	2.22	2.23	2.45	1.02	2.77	2.49
**9**	5	12	25	1.31	1.4	2.03	2.62	1.88	1.93	2.65	3.57
**10**	5	12	25	1.25	0.69	1.58	1.97	1.83	1.83	2.60	3.42
**11**	5	48	75	2.01	2.57	1.69	1.3	2.23	2.23	2.64	1.81
**12**	5	48	75	1.66	1.57	2.08	1.49	2.27	2.22	2.68	1.75
**13**	5	30	50	2.08	2.07	1.90	1.92	2.50	2.54	2.64	2.63
**14**	5	30	50	2.10	2.12	1.95	1.97	2.54	2.52	2.62	2.61
**15**	5	30	50	2.07	2.09	1.91	1.90	2.52	2.51	2.60	2.64

Supplementary TABLE S3 Estimated regression coefficients, standard errors and significance of Box-Behnken models.

Carbon source	Factor	Coefficient	Standard Error	*t*-value	*P*-value	Confidence level (%)
**Full wheat grains**	**Intercept**	0.6307	0.2586	25.1351	0.0261	97.39
	X1	-0.3630	0.1584	3.1573	0.0196	98.04
	X1X1	0.0332	0.1584	4.0256	0.0069	99.31
	X2	-0.0274	0.2240	-1.0047	0.3538	64.62
	X2X2	0.0003	0.2240	0.2233	0.8307	16.93
	X3	0.0701	0.2758	0.7895	0.8568	14.32
	X3X3	-0.0007	0.3167	1.0261	0.7601	23.99
	X1X2	0.0162	0.2331	1.1261	0.3031	69.69
	X2X3	-0.0001	0.2331	-1.3406	0.2286	77.14
	X1X3	-0.0004	0.2331	-5.6306	0.0013	99.87
**Half crushed wheat grains**	**Intercept**	0.9420	0.3530	34.2812	0.0404	95.96
	X1	0.2171	0.2161	3.4121	0.0143	98.57
	X1X1	-0.0189	0.2161	3.4121	0.0143	98.57
	X2	0.0281	0.3057	4.2529	0.0054	99.46
	X2X2	0.0000	0.3057	-0.0818	0.9375	6.25
	X3	0.0075	0.3564	-0.1324	0.8642	13.58
	X3X3	0.0000	0.4323	-2.4289	0.7621	23.79
	X1X2	-0.0081	0.3182	2.6323	0.0389	96.11
	X2X3	-0.0001	0.3182	-1.6108	0.1583	84.17
	X1X3	-0.0005	0.3182	-0.4322	0.6807	31.93
**Wheat flour 82%**	**Intercept**	0.2259	0.1298	1.7404	0.1568	84.32
	X1	-0.0920	0.0417	-2.2059	0.0920	90.8
	X1X1	0.0262	0.0039	6.7895	0.0025	99.75
	X2	0.1150	0.0060	19.2097	0.0000	100
	X2X2	-0.0016	0.0001	-17.8295	0.0001	99.99
	X3	0.0575	0.0021	27.6559	0.0000	100
	X3X3	-0.0005	0.0000	-28.0102	0.0000	100
	X1X2	-0.0191	0.0016	-12.1391	0.0003	99.97
	X2X3	0.0002	0.0000	13.5871	0.0002	99.98
	X1X3	-0.0002	0.0002	-1.1043	0.3314	66.86
**Wheat flour 72%**	**Intercept**	2.1591	0.1298	16.6326	0.0001	99.99
	X1	0.1403	0.0417	3.3621	0.0283	97.17
	X1X1	-0.0145	0.0039	-3.7546	0.0199	98.01
	X2	0.0223	0.0060	3.7208	0.0205	97.95
	X2X2	-0.0001	0.0001	-1.3001	0.2634	73.66
	X3	0.0135	0.0021	6.5054	0.0029	99.71
	X3X3	-0.0002	0.0000	-8.5940	0.0010	99.9
	X1X2	-0.0136	0.0016	-8.6246	0.0010	99.9
	X2X3	0.0001	0.0000	5.3099	0.0060	99.4
	X1X3	0.0004	0.0002	2.8712	0.0454	95.46

Abbreviations: *X1*, yeast extract (g/l); *X2*, carbon source (g/l); and *X3*, medium volume (ml).
